# HAM-net national patient registration system reveals details of how Japanese patients with HTLV-1-associated myelopathy/tropical spastic paraparesis progress over time

**DOI:** 10.1186/1742-4690-12-S1-O2

**Published:** 2015-08-28

**Authors:** Ariella Coler-Reilly, Naoko Yagishita, Tomoo Sato, Natsumi Araya, Miho Ishikawa, Mikako Koike, Yumi Saito, Hiroko Suzuki, Yoshihisa Yamano, Ayako Takata

**Affiliations:** 1 Department of Rare Diseases Research, Institute of Medical Science, St. Marianna University School of Medicine, Kawasaki, Kanagawa, Japan; 2 Department of Preventive Medicine, St Marianna University School of Medicine, Kawasaki, Kanagawa, Japan; 3 Intractable Disease Consultation, St. Marianna University School of Medicine, Kawasaki, Kanagawa, Japan

## 

In order to better understand and manage HTLV-1 associated myelopathy/tropical spastic paraparesis (HAM/TSP), a rare virus-induced neurodegenerative disease, we have established a registration system known as HAM-net to gather data from patients all over Japan. In this prospective epidemiological study, we analyzed the data from the 297 registered patients who were available to be interviewed once upon registration and again one year later. On average, these patients were 63.2 years old, had experienced their first symptoms at age 44.2, and were diagnosed at age 51.3. More patients were female (74.1%) than male (25.9%). During the one-year interval between surveys, the average Health Assessment Questionnaire Disability Index (HAQ-DI) score rose significantly (P<0.001), indicating noticeably declining health. The average Osame Motor Disability Score (OMDS) also worsened (P<0.001), rising from 5.9±2.3 to 6.2±2.4. While the majority of patients (81%) experienced no change in OMDS, 53 patients (18%) worsened, and only 4 patients (1%) improved. Importantly, when compared to patients given other treatments, those treated continuously with oral steroids were less likely to experience a rise in OMDS. Fifty-four of the patients were deemed “rapid progressors,” defined as a patient who progressed from the onset of motor symptoms to at least OMDS 5 within two years, and 15 of those were “very rapid progressors,” defined as those who reached at least OMDS 6. Both rapid progressors and very rapid progressors exhibited several statistically significant traits compared to the other patients: older when motor symptoms first manifested, older when first diagnosed, higher OMDS, higher HAQ-DI score, shorter gap between disease onset and diagnosis, and shorter disease duration from onset to present day. We will continue registering new patients and analyzing changes in their health over time.

